# Does Social and Economic Disadvantage Predict Lower Engagement with Parenting Interventions? An Integrative Analysis Using Individual Participant Data

**DOI:** 10.1007/s11121-022-01404-1

**Published:** 2022-07-23

**Authors:** Vashti Berry, G. J. Melendez-Torres, Nick Axford, Ulf Axberg, Bram Orobio de Castro, Frances Gardner, Maria Filomena Gaspar, Bjørn Helge Handegård, Judy Hutchings, Ankie Menting, Sinéad McGilloway, Stephen Scott, Patty Leijten

**Affiliations:** 1 University of Exeter, EX1 2LU Exeter, UK; 2 University of Plymouth, Plymouth, UK; 3 VID Specialized University, Oslo, Norway; 4 University of Amsterdam, Amsterdam, Netherlands; 5 University of Oxford, Oxford, UK; 6 University of Coimbra, Coimbra, Portugal; 7 UiT The Arctic University of Norway, Tromsø, Norway; 8 Bangor University, Bangor, UK; 9 Utrecht University, Utrecht, Netherlands; 10 Maynooth University, Maynooth, Ireland; 11 King’s College London, London, UK

**Keywords:** Parenting programs, Socioeconomic status, Social disadvantage, Engagement, IPD meta-analysis

## Abstract

There is a social gradient to the determinants of health; low socioeconomic status (SES) has been linked to reduced educational attainment and employment prospects, which in turn affect physical and mental wellbeing. One goal of preventive interventions, such as parenting programs, is to reduce these health inequalities by supporting families with difficulties that are often patterned by SES. Despite these intentions, a recent individual participant data (IPD) meta-analysis of the Incredible Years (IY) parenting program found no evidence for differential benefit by socioeconomic disadvantage (Gardner et al. in Public Health Resesearch 5, 1–144, 2017). However, it did not examine whether this was influenced by engagement in the intervention. Using intervention arm data from this pooled dataset (13 trials; *N* = 1078), we examined whether there was an SES gradient to intervention attendance (an indicator of engagement). We ran mixed-effects Poisson regression models to estimate incidence rate ratios (IRRs) for program attendance for each of five (binary) markers of SES: low income; unemployment; low education status; teen parent; and lone parent status. The multilevel structure of the data allowed for comparison of within-trial and between-trial effects, including tests for contextual effects. We found evidence that low SES was associated with reduced attendance at parenting programs—an 8–19% reduction depending on the SES marker. However, there was no evidence that this association is impacted by differences in SES composition between trials or by the attendance levels of higher-SES families. The findings underscore the importance of developing and prioritizing strategies that enable engagement in parenting interventions and encourage program attendance by low-SES families.

**Supplementary Information:**

The online version contains supplementary material available at 10.1007/s11121-022-01404-1.

## Introduction

It is well established that health is influenced by a broad range of social and economic conditions, or social determinants, which by the nature of their distribution in the population give rise to inequalities in life chances, disease experience, and life expectancy (Public Health England (PHE), 2017; World Health Organisation (WHO), 2019). Simply put, the poorer one’s socioeconomic status (SES) in society, the poorer one’s health outcomes—mortality, morbidity, risk behaviors, and mental health—are likely to be.

The need to reduce health inequalities (i.e., to diminish outcome gaps between rich and poor) is widely recognized. There is a broad consensus that doing so requires investing in multi-sectoral policies that address social, economic, environmental, and commercial determinants. Part of this involves developing proportionate-universal^1^ prevention responses rather than concentrating interventions only on poorer segments of society (Marmot, 2014; Marmot Review Team, 2010). Targeted approaches can stigmatize offers of support and often do not identify all children and families in need of additional help (Hurt et al., 2018; Hutchings et al., 2013). Addressing health inequalities also involves supporting parents to give children the best start in life, including through the delivery of evidence-based parenting programs in local statutory and non-profit services (Jensen et al., 2013; PHE, 2014; Saunders et al., 2017; United Nations Office on Drugs and Crime (UNODC), 2009; WHO, 2019).

Indeed, there is a considerable evidence base for the effectiveness of parenting programs in reducing parents’ and children’s emotional and behavioral difficulties and promoting health outcomes (e.g., Barlow et al., 2010; Furlong et al., 2012; Leijten et al., 2018; Rayce et al., 2017; Zwi et al., 2011). Many such programs are listed in the growing number of online registries hosted by government and non-governmental organizations and designed to inform investment decisions by policy makers and commissioners. However, if goals of public health policy and intervention include reducing health inequalities, thereby ensuring that unintended inequalities do not emerge as a result of differential access or uptake of support, then further research is needed to interrogate the differential benefit of interventions by SES.

SES is a multi-faceted concept that relates to the position of an individual, family, or area/neighborhood in society, with respect to education and occupational level, income, and social resources (Lynch & Kaplan, 2000). There is no consensus on operationalizing SES but common methods for measuring SES in individuals and families include distinguishing between “high” and “low” classes of monthly/annual income and eligibility for state-provided welfare benefit; occupational type (e.g., unemployed; manual v. non-manual roles); and highest level of education attained (e.g., primary, secondary, or post-secondary) (Conway et al., 2019). In addition, studies have sought to compare differences in social capital in the form of (security of) housing type and social support or indicators reflecting inequalities in social assets or demographics (e.g., lone parent, teen/young parent). Composites of these indicators are common and studies may be based on objective data or reports of a person’s self-perceived status relative to their peers. At an area/neighborhood level, social epidemiologists typically rely on deprivation indices (e.g., Townsend et al., 1988), mean levels of household income, and/or aspects of the social or physical environment such as the availability of green space, public transport, and services.

Evidence on the relative effectiveness of parenting support for low-SES families is patchy, with mixed results. A recent meta-synthesis of systematic reviews of effective parenting interventions found that only half of the included reviews considered social inequalities in health and those that did were “modest and reductive” in their approach (Pierron et al., 2018, p. 25). Where data is available, patterns are not clear. Two meta-analyses of parent training programs (Lundahl et al., 2006; Reyno & McGrath, 2006) found that socially disadvantaged families benefitted less from the intervention, compared to more advantaged families, while a third review of group-based programs (Furlong et al., 2012) found no differences in impact by SES. Patterns may also be inconsistent; Gardner et al. (2009) found that while children of parents with low educational attainment benefitted most from a parenting intervention for children with behavioral difficulties (the Family Check-Up), positive impacts were also more likely in two-parent families than lone parent households. Thus, the intervention may widen health inequalities if the needs of single-parent families are not better addressed. It would be concerning if this pattern (i.e., effective interventions benefitting better-off participants disproportionately) is replicated more widely.

To explore this issue, a recent individual participant data (IPD) meta-analysis pooled data from 14 European trials of one well-established parenting program (the Incredible Years (IY)), including data from 1799 families with children aged 2–10 years (Gardner et al., 2017, 2019). The study explored moderators of program effectiveness, including whether there were differential benefits for groups marked by social disadvantage (Gardner et al., 2019). It found no evidence that any indicator of socioeconomic disadvantage (or membership of an ethnic minority group) affected program benefit; the program is as likely to be beneficial for socially disadvantaged families as it is for non-disadvantaged. While partially encouraging, in that IY did not appear to widen health inequalities even if it did not reduce them during the study period, the IPD study did not explore whether engagement with the intervention differed by (latent) social determinants. Thus, the study concluded that low-SES families were equally likely to benefit from being allocated to the intervention, regardless of unexplored and potentially differential engagement. Given the association between engagement in parenting interventions and their effectiveness identified in some trials (e.g., Baydar et al., 2003; Ros et al., 2016), it is important to understand whether attendance was indeed differential by SES. Thus, the present study aimed to examine, using a large pooled sample data from multiple trials, whether there is an SES gradient to parenting program attendance, as an indicator of intervention engagement.

### Intervention Engagement

Engagement in parenting programs can be understood in four ways: recruitment—approaching parents in the target population and securing an indication of their intention to attend; enrolment—recruited parents who turn up at least once; retention—the extent of parents’ attendance at sessions during the program; and active involvement or participation in the program sessions (Hackworth et al., 2018). This study is concerned with the second and third forms of intervention engagement, that is, participants’ enrolment in a parenting program and attendance at program sessions, and the extent to which these are associated with social disadvantage. Several studies have demonstrated the importance of attendance and engagement in determining the benefits gained from parenting interventions (e.g., Baydar et al., 2003; Gross et al., 2009; Joseph et al., 2019) although not all have translated into better child outcomes (e.g., Weeland et al., 2017). In addition, several studies have demonstrated that between a quarter and a third of parents who begin parenting programs do not continue to attend or complete them (Lindsay et al., 2008; Scott & Dadds, 2009). Moreover, many parents who are referred do not enroll in the offered intervention, not attending a single session (Axford et al., 2012; Baker et al., 2011).

There are myriad reasons for poor program enrolment and attendance, many of which intersect with social disadvantage. Some concern accessibility. These include competing demands on parents’ time and resources and practical barriers such as lack of transport or childcare (Hutchings et al., 2004). The frequency and timing of group sessions may also discourage attendance; conventional practice is to hold groups during the day which may not accommodate parents without the flexibility to consistently attend appointments during business hours (Hodgkinson et al., 2017). Social isolation and a lack of peer support can also reduce motivation to attend (Boag-Munroe & Evangelou, 2012). Other barriers relate more to the acceptability of services, for example, the social stigma attached to attending parenting courses and fear of being labeled a “bad parent” (Dempster et al., 2013; Furlong & McGilloway, 2015). Then there are awareness barriers, reflecting a lack of knowledge about the availability of local support services or a failure to appreciate the need for support (Pote et al., 2019). Parents’ education levels may influence their perception of need for services. A five-country study of Early Childhood Education and Care (ECEC) services, which incorporate parenting programs, found that low income, low maternal education, and having more than one child were associated with reduced use of ECECs (Petitclerc et al., 2017). Consequently, families with the greatest potential to benefit from participation may be the least likely to enroll in and attend interventions (Porterfield & McBride, 2007).

Gardner et al. (2017) suggest that two aspects of the IY program may help to enhance its suitability for a diverse range of families (including those from low SES backgrounds), including “its inbuilt collaborative and flexible style, and the efforts it makes to remove barriers to access for families, including provision of child care and meals during the group sessions” (p. 87). These kinds of approaches, and the discrete strategies employed (see below), are widely regarded as important for engaging parents and clearly address identified barriers to engagement, although evidence for their impact—particularly on different aspects of engagement—is limited owing to a lack of research (Finan et al., 2018; Hackworth et al., 2018; Pote et al., 2019).

Given these program characteristics and the attention to program fidelity in clinical trials, the lack of equity-generating impact across the IY trials is surprising at face value. However, it may be explained by the individual and community-level socioeconomic conditions that potentially influenced families’ engagement. Specifically, service-level strategies to support participant enrolment and attendance varied considerably across the 14 trials and are likely to vary even more widely in real-world implementation. These included home visits by facilitators to meet families before the intervention began (trials #2, #5, #6, #8, #9, #13) as well as catch-up contacts between sessions for parents who missed a weekly session (#3, #6, #9, #13); childcare for young children during the sessions (#1, #2, #3, #4, #6, #7, #8, #9, #13, #14); transport or financial aid, where required, for journeys to/from the program venue (#2, #4, #5, #6, #7, #14)^2^; provision of light refreshments (#3, #9) or meals during the sessions (#1, #4, #5, #7); and rewarding parents with small gifts for attendance (#3, #5). If low SES reduced the likelihood of attendance, but low-SES families nevertheless benefitted to the same degree as non-disadvantaged families, there is scope to reduce inequalities in outcomes by addressing factors that might increase the engagement of socially disadvantaged families.

### Integrative Data Analysis

Adequately exploring whether low SES predicts poorer engagement with parenting interventions requires a sufficiently large sample of families and sufficient variation in SES and program attendance rates (Curran & Hussong, 2009). Additionally, a degree of consistency in the parenting intervention under investigation is needed to ensure that differential engagement is not a function of differences in program approaches. By integrating individual family-level data across several trials of the same intervention (IY), the IPD pooled dataset (Gardner et al., 2017) provides both the sample and the variation needed to have the statistical power to precisely estimate these relationships. In integrative data analysis, data from different samples collected as part of multiple independent studies are analyzed together, taking the multilevel structure of the data (individuals nested in trials) into account (Curran & Hussong, 2009). Such analysis is increasingly used in prevention research and comes with several challenges (e.g., harmonizing scores from different measures used in different studies and the ability and willingness of researchers to share their data) which are increasingly being addressed within the field (e.g., Perrino et al., 2013). These advances increase the feasibility of integrative data analysis and its contributions to prevention science.

## Methods

### Design and Procedure

We obtained the pooled dataset of individual participant data from 14 trials conducted between 1995 and 2013 to investigate the effects of the IY parenting program for children aged 2–10 years across six countries in Europe. The study protocol for integration is published at www.spi.ox.ac.uk/parentingIPD as are the details of how the data were harmonized (Gardner et al., 2017; Leijten et al., 2018). All of the trials were conducted independently of the US-based program developer (Carolyn Webster-Stratton) and all were eligible for inclusion in the present study (details in Table 1). However, only 13 trials reported program attendance data; hence, participants in the current study are the 1078 intervention families in these 13 trials.

**Table 1:** Included trials

**Trial**	**Lead author (year)**	**Country**	**Trial** ***N***	**IY Arm** ***N***	**# sessions offered**	**% sessions attended**	**Intervention participants attending zero sessions;** ***N*** **(%)**
#1	Larsson (2009)	Norway	75	47	12–13	33–100 (*M* = 91; *SD* = 14)	0 (0%)
#2	Axberg (2012)	Sweden	62	38	–	–	–
#3	Seabra-Santos (2016)	Portugal	124	68	14	7–100 (*M* = 78; *SD* = 25)	0 (0%)
#4	McGilloway (2012)	Ireland	149	103	12–14	0–100 (*M* = 59; *SD* = 39)	14 (14%)
#5	Menting (2014)	Netherlands	99	74	12	0–100 (*M* = 42; *SD* = 37)	25 (34%)
#6	Leijten (2017)	Netherlands	156	109	12–18	0–100 (*M* = 68; *SD* = 29)	3 (5%)
#7	Hutchings (2007)	Wales	153	104	12	0–100 (*M* = 65; *SD* = 26)	12 (12%)
#8	Hutchings (2017)	Wales	103	70	12	0–100 (*M* = 61; *SD* = 35)	7 (10%)
#9	Morpeth (2017)	England	161	110	12	0–100 (*M* = 47; *SD* = 42)	37 (35%)
#10	Scott (2010b)	England	112	61	28	0–100 (*M* = 57; *SD* = 35)	4 (7%)
#11	Scott (2010a)	England	174	88	18	0–100 (*M* = 27; *SD* = 32)	30 (34%)
#12	Scott (2014)	England	214	106	12	0–100 (*M* = 71; *SD* = 28)	0 (0%)
#13	Gardner (2006)	England	76	48	14	0–100 (*M* = 55; *SD* = 38)	10 (23%)
#14	Scott (2001)	England	141	90	11–19	7–100 (*M* = 76; *SD* = 24)	0 (0%)

### The Intervention

Families in the intervention condition were offered the IY parenting program, a weekly, facilitated group-based intervention grounded in social learning and attachment theory (Webster-Stratton & Reid, 2010). The number of sessions offered ranged from 11 to 28 across trials, depending on the timing and context of the trials; older versions and prevention (versus treatment) versions of IY include fewer sessions, and two trials (#10 and #11) included sessions on helping parents read with their children.

### Measures

#### Program Engagement

The dataset captures, for each family in the 13 included trials, the number of parenting program sessions attended (absolute dosage, which ranged from 0 to 22—continuous variable) and the proportion of sessions attended as a function of the program length (relative dosage). Relative dosage is used in the current study as an indicator of participant engagement with the program, with higher attendance indicating greater engagement.

#### Socioeconomic Status

The pooled IPD of 14 trials contains five harmonized variables (Gardner et al., 2019) considered to be indicators of SES:

1. Low income: indicators were defined as receiving income-related financial benefits (11 trials), scoring below Hollingshead Index’s (Hollingshead, 2011) low-SES threshold (1 trial) or living in social housing (2 trials).
2. Low education: highest educational level of parent was dichotomized using UNESCO ISCED-11 categories, where “low education” = primary/lower secondary, and “high” = upper secondary/degree-level education or above.
3. Lone parenthood: primary parent lives without partner/spouse.
4. Teen parenthood: parent < 20 years at index child’s birth.
5. Unemployment: no parent in household employed.

### Analytic Strategy

Our objective was to establish whether there is an SES gradient to parenting program attendance. Because our dependent variable was count distributed, i.e., number of sessions attended, we ran mixed-effects Poisson regression models with participants clustered within trials to estimate incidence rate ratios (IRRs) for session attendance for each of the five (binary) markers of SES. Length of observation differed by trial and we thus adjusted models to account for differential exposure, using the number of offered sessions as the exposure variable (i.e., coefficient fixed to 1). Within-study data missingness was handled by maximum likelihood estimation and statistical analyses were conducted, using Stata (v16), in three phases for each indicator:

1. First, we estimated the “unconditional” relationship between each SES indicator and attendance, asking the question: what is the impact within a trial of low SES on attendance for a given parent? (model 1). In this model, we entered the individual-level SES indicator as the only predictor of attendance.
2. Second, because the multilevel structure of the data allows for comparison of within-trial and between-trial effects, we parsed individual and contextual effects (i.e., the effect of trial-level average values of SES indicators over and above individual values of the SES indicator) by using a trial-level estimate of the SES indicator and an individual-level, trial-centered value of the SES indicator (model 2). This model jointly answers three questions: what is the impact within a trial of low SES on attendance for a given parent (within-trial effects); what is the impact of trial-level SES on trial-level attendance (between-trial effects); and does the SES composition of a trial sample have impacts on attendance that go beyond individual parents’ SES (contextual effects)?
3. Third, we covaried trial-level intercepts with the individual-level, trial-centered value of the SES indicator to account for relationships between “intercept” level of attendance and SES gradients in attendance (model 3). This random slopes analysis allows each group line to have a different slope, and in this case related the attendance for higher-SES families in each trial to the steepness of the SES slope. This model answers the three questions of model 2 with an additional test for covariance that checks whether the within-trial relationship of SES with attendance is stronger or weaker when attendance by higher-SES families is higher. We were not able to estimate cross-level interactions between individual-level and trial-level SES in this last step, because the models did not converge.

## Results

There was variation across the trials both in attendance and the degree of low SES in the sample. Table 1 indicates that in four trials (#1, #3, #12, #14) all enrolled participants attended at least one program session. In contrast, around a third of participants in three trials (#5, #9, #11) did not attend even one session. Mean percentage attendance (relative dosage) ranged from 27% (trial #11) to 91% (#1), and across trials parents attended on average 59% of the sessions (range *M*_percent sessions attended_ = 27 to 91%). Table 2 provides summary statistics for the SES indicators at both trial level and within the intervention arm. Some trials over-sampled families from disadvantaged areas (#4, #5, #6, #7, #12) while others had relatively low levels of social disadvantage (#1, #3) compared to the national average. Although there was a significant association between different indicators of SES (*r*s = 0.178 to 0.555), there was by no means perfect convergence (Online Resource Table S1). Some indicators were not available in all trials (e.g., unemployment); the most commonly reported classification of low SES across trials was low income (*M*_all trials_ = 58%).

**Table 2:** Summary statistics of SES indicators for each trial sample

**Trial**	**Low income mean (of** ***N*)**	**Un-employed mean (of** ***N*)**	**Teen parent mean (of** ***N*)**	**Lone parent mean (of** ***N*)**	**Low education mean (of** ***N*)**
#1 Trial-level IY Arm only	25% (63) 24% (42)	13% (71) 13% (47)	10% (68) 11% (45)	33% (66) 34% (41)	17% (75) 13% (47)
#2 Trial-level IY Arm only	41% (56) 43% (35)	2% (59) 3% (38)	2% (53) 0% (33)	33% (57) 31% (35)	19% (62) 18% (38)
#3 Trial-level IY Arm only	0% (124) 0% (68)	1% (118) 2% (64)	5% (122) 5% (66)	20% (124) 16% (68)	21% (124) 24% (68)
#4 Trial-level IY Arm only	47% (148) 45% (102)	47% (142) 46% (97)	9% (148) 10% (103)	38% (149) 38% (103)	44% (149) 43% (103)
#5 Trial-level IY Arm only	93% (99) 92% (74)	87% (68) 90% (50)	16% (91) 12% (68)	71% (99) 70% (74)	73% (99) 70% (74)
#6 Trial-level IY Arm only	74% (156) 76% (109)	–	3% (156) 2% (109)	7% (156) 7% (109)	54% (156) 57% (109)
#7 Trial-level IY Arm only	80% (153) 80% (104)	–	28% (153) 29% (104)	43% (153) 48% (104)	48% (153) 47% (104)
#8 Trial-level IY Arm only	56% (103) 60% (70)	40% (90) 43% (60)	41% (99) 43% (68)	38% (103) 41% (70)	39% (103) 40% (70)
#9 Trial-level IY Arm only	63% (160) 62% (109)	52% (161) 54% (110)	14% (159) 14% (108)	37% (160) 38% (109)	44% (161) 46% (110)
#10 Trial-level IY Arm only	44% (105) 47% (58)	28% (111) 33% (61)	10% (110) 12% (60)	28% (109) 31% (58)	36% (112) 34% (61)
#11 Trial-level IY Arm only	44% (166) 43% (88)	25% (160) 24% (83)	11% (155) 11% (82)	41% (162) 42% (85)	30% (174) 27% (88)
#12 Trial-level IY Arm only	81% (201) 69% (95)	29% (207) 28% (101)	9% (193) 9% (99)	32% (208) 33% (104)	45% (214) 42% (106)
#13 Trial-level IY Arm only	64% (75) 64% (47)	61% (71) 63% (43)	18% (74) 13% (46)	47% (76) 50% (48)	82% (76) 83% (48)
#14 Trial-level IY Arm only	58% (108) 60% (68)	41% (135) 47% (87)	17% (127) 16% (81)	45% (87) 48% (62)	87% (141) 82% (90)

We estimated IRRs for the five SES predictors on parenting program attendance (Table 3). In our first stage models, all indicators significantly predicted less attendance, with IRRs ranging from an 8% (lone parent status) to a 19% (low education) reduction in program attendance. It is notable that, after parent educational attainment, economic and occupational markers of low SES (e.g., low income and unemployment) appeared to have greater impact on attendance than sociodemographic markers (e.g., lone parent and teen parent). Our second stage models estimated contextual effects for low SES on attendance, that is, whether trials in our IPD with samples with higher levels of disadvantage demonstrated worse attendance over and above the impact of individual-level disadvantage. There was no systematic evidence of contextual effects, although this finding is caveated by the small level 2 sample size of 13 trials.

**Table 3:** Mixed-effects Poisson regression models by SES/disadvantage indicator

	**Model 1** **IRR (95% CI)**	**Model 2** **IRR (95% CI)**	**Model 3** **IRR (95% CI)**
**Family has—or is at risk for—low income**			
Intercept	0.63 (0.53, 0.75)	0.68 (0.44, 1.05)	0.67 (0.43, 1.05)
Predictor	0.84 (0.80, 0.88)*		
Centered predictor		0.84 (0.80, 0.88)*	0.84 (0.76, 0.94)*
Trial-level predictor		0.74 (0.35, 1.53)	0.75 (0.36, 1.55)
Random intercept variance	0.10 (0.04, 0.21)	0.10 (0.04, 0.21)	0.10 (0.05, 0.22)
Random slope variance			0.03 (0.01, 0.08)
Covariance: random intercept, random slope			0.01 (−0.02, 0.04)
**Unemployed carer—there is no employed parent in the household**			
Intercept	0.59 (0.48, 0.72)	0.68 (0.47, 1.00)	0.68 (0.47, 1.00)
Predictor	0.88 (0.84, 0.93)*		
Centered predictor		0.89 (0.84, 0.94)*	0.90 (0.82, 1.00)
Trial-level predictor		0.61 (0.27, 1.38)	0.62 (0.27, 1.41)
Random intercept variance	0.11 (0.05, 0.26)	0.10 (0.04, 0.24)	0.10 (0.04, 0.24)
Random slope variance			0.02 (0.00, 0.07)
Covariance: random intercept, random slope			0.01 (−0.03, 0.04)
**Teenage parent—primary parent/s was younger than 20 at birth of target child**			
Intercept	0.59 (0.50, 0.69)	0.58 (0.44, 0.77)	0.58 (0.43, 0.77)
Predictor	0.90 (0.84, 0.96)*		
Centered predictor		0.90 (0.84, 0.96)*	0.88 (0.77, 1.00)
Trial-level predictor		1.01 (0.20, 5.09)	0.99 (0.19, 5.20)
Random intercept variance	0.09 (0.04, 0.20)	0.09 (0.04, 0.20)	0.09 (0.04, 0.20)
Random slope variance			0.04 (0.01, 0.13)
Covariance: random intercept, random slope			0.00 (−0.04, 0.05)
**Lone parent—the primary parent does not live with a partner or spouse**			
Intercept	0.59 (0.50, 0.70)	0.76 (0.49, 1.18)	0.79 (0.51, 1.22)
Predictor	0.92 (0.88, 0.97)*		
Centered predictor		0.92 (0.88, 0.97)*	0.93 (0.86, 1.00)
Trial-level predictor		0.48 (0.16, 1.39)	0.43 (0.15, 1.26)
Random intercept variance	0.10 (0.04, 0.21)	0.09 (0.04, 0.19)	0.09 (0.04, 0.19)
Random slope variance			0.01 (0.00, 0.04)
Covariance: random intercept, random slope			0.01 (−0.01, 0.03)
**Low education—highest educational level of primary**			
Intercept	0.63 (0.53, 0.75)	0.59 (0.40, 0.89)	0.58 (0.43, 0.80)
Predictor	0.81 (0.77, 0.85)*		
Centered predictor		0.81 (0.77, 0.85)*	0.80 (0.70, 0.91)*
Trial-level predictor		0.94 (0.41, 2.14)	0.96 (0.54, 1.70)
Random intercept variance	0.10 (0.05, 0.22)	0.10 (0.05, 0.22)	0.10 (0.05, 0.23)
Random slope variance			0.05 (0.02, 0.13)
Covariance: random intercept, random slope			0.06 (0.00, 0.12)*

Model 1: IRRs for session attendance (uncentered)

Model 2: centered predictor model

Model 3: centered predictor model adjusting for baseline (intercept) values

^*^Significant at the 0.05 significance level

Finally, our checks on the intercept slope relationship revealed no evidence of covariance for four of the SES indicators (model 3); that is, levels of non-attendance among higher-SES families in trials did not appear to relate to the social gradient for non-attendance. Low education was the exception to that finding, where higher levels of attendance by higher-SES families were related to weaker SES (educational) gradient differences (*p* = 0.03).

To establish (post hoc) the credibility of the hypothesis that more attendance is linked to greater effectiveness, we estimated a random intercept model predicting treatment outcome (child behavior using the Eyberg Child Behavior Inventory; ECBI, Eyberg & Ross, 1978) at follow-up, with a trial-level predictor as percentage of sessions attended, and two parent-level predictors: baseline ECBI and trial-centered percentage of sessions attended. Both percentage of sessions attended predictors were rescaled so a one-unit change in the predictor corresponded to a change in attendance of 10 percentage points. This model revealed that an increase in attendance of 10 percentage points at the parent level was linked to a decrease in ECBI of 0.69 points (95% CI [−1.34, −0.05]). Trial-level percentage of sessions attended was linked to a similar decrease of 0.53 points but this was not significant (*p* = 0.591). While this decrease may seem small, it equates to ~ 2% of 1 *SD* on the ECBI. Translating this into intervention benefits, parents who attend 50% more sessions show an increase in intervention benefit of Cohen’s *d* = 0.10, and attending twice as many sessions leads, on average, to an increase in intervention benefit on the ECBI of *d* = 0.20.

## Discussion

Intervention engagement is essential for intervention benefits. It is important, therefore, to identify those aspects of socioeconomic status that hinder intervention engagement and by so doing, better understand the extent to which preventive interventions can reduce health inequalities. We used individual participant data meta-analysis to explore the influence of low socioeconomic status on intervention attendance, as an indicator of engagement. By integrating data across multiple trials of a common intervention, we identified significant individual differences in attendance rates by SES. All SES indicators were associated with a reduction in attendance, with low educational level resulting in nearly a 20% reduction. While it may not be surprising that socioeconomically disadvantaged families attended fewer sessions, its implications are significant, especially in the light of earlier findings that these families did not benefit differently in terms of reduced children’s conduct problems (Gardner et al., 2019). Specifically, measures to improve parents’ attendance have the potential to boost the impact of parenting interventions on the most disadvantaged, thereby reducing health inequalities.

That said, perhaps contrary to expectations given evidence from other studies of an effect of neighborhood deprivation on health (e.g., Algren et al., 2015; Stafford & Marmot, 2003; Zhu et al., 2022), trials with higher average levels of SES did not demonstrate steeper social gradients. This means that attendance was driven by the individual markers of SES, rather than by the concentration of social disadvantage in the sample. Our covariance checks also confirmed that the attendance rates of higher-SES families did not significantly influence these observed social gradients. In other words, trials with better attendance in higher-SES families still demonstrated the same associations with low SES on attendance as those with poorer attendance by higher-SES families. The exception to this finding was the low education marker of SES, where higher average levels of attendance by those with high education in trials were related to a weaker (i.e., better) SES gradient of low education on attendance. Collectively, these findings might suggest that the additional “wraparound” support provided by the trials/services, in the form of meals, childcare, and transport, for example, may be a necessary but not sufficient means of weakening the social gradient of attendance.

Considering the complex relationship between SES and parenting behaviors (Roubinov & Boyce, 2017), it is likely that services will need to do more (e.g., in terms of level of effort, resources, and creative thinking) to support the engagement of socially disadvantaged families in parenting programs. First, additional effort is needed where there are parallel concerns about poor parental mental health. Although this is known to be associated with higher program effects (Leijten et al., 2020), it is also linked to lower program enrolment and attendance, for instance because parents lack confidence, feel judged or blamed by practitioners for their child’s behavior, have negative perceptions of services or their own potential for change, or attribute difficulties to external rather than internal factors within their control (Pote et al., 2019). Programs and services need to address these issues. A pre-intervention approach that targets such beliefs can change parents’ perception of the need and strengthen their sense of parenting self-efficacy (Shepard et al., 2012), in that instance increasing enrolment in the IY program. Parenting program practitioners also need to be encouraging, non-judgmental, and patient so that parents feel validated and involved throughout (e.g., Wilson et al., 2018). The ways in which practitioners and other staff interact with parents at the point of access to the intervention also require particular thought (e.g., Eisner & Meidert, 2011). A recent study using conversation analysis of recordings of parenting practitioners’ initial engagement with parents demonstrated that most calls focused on practical arrangements of the service, rather than understanding parents’ needs and building relationships prior to working together. However, when practitioners made “interactional space” for parents to recount their situation, it created opportunities to orient parents towards the service, potentially influencing the parents’ decision to engage (Symonds, 2018). Critically, these engaged conversations took three times longer than administratively styled calls, carrying implications for service planning.

Second, wraparound supports to enable attendance need to be integral—as opposed to optional add-ons—to parenting programs. While they are a recommended part of (IY) parenting support provision, they were provided to differing degrees in the trials synthesized in the IPD and are often overlooked in real-world implementation, due to perceived organizational burden and/or limited financial resources (Axford et al., 2012; Hickey et al., 2021). If the health benefit for low-SES families demonstrated in the IPD analysis was influenced by the high levels of wraparound support offered to families during the clinical trials, it is plausible that such families may be less able to attend the intervention in the context of reduced support for enrolment and retention in real-world services. This would produce a regressive differential impact on outcomes (poorer outcomes for more disadvantaged children). Future research should focus on the barriers to and enablers of implementation through a social gradient lens, and seek to understand how best to integrate gold-standard interventions, like IY, into existing community services and systems in a way that increases engagement for those most disadvantaged. Implementation success is likely to depend on complex “person-program-contextual interactions” (Hickey et al., 2021), making a one-size-fits-all approach undesirable.

Third, parenting programs need to flex to meet parents’ needs and empower them to make choices; additional components or brief, pre-intervention work may improve treatment motivation and engagement (Berkel et al., 2021; Nock & Kazdin, 2005; Shepard et al., 2012; Stormshak et al., 2021). A recent systematic review of strategies used to increase engagement in preventive parenting programs found a lack of consistent evidence for individual or family-level predictors of parent engagement; only the severity of child’s difficulties was related to increased likelihood of enrolment and, even then, this did not predict ongoing engagement with the intervention (Finan et al., 2018). A meta-analysis was not possible but the authors suggest that engagement strategies underpinned by established theories, such as the Theory of Planned Behavior (TPB; Ajzen, 1991) and the Health Belief Model (HBM; Rosenstock, 1974), should help to increase parents’ engagement in preventive parenting programs. These suggest that while reducing perceived barriers to participation, such as providing childcare for young children or personalizing engagement contacts, may be useful enrolment strategies, they are unlikely to be sufficient to sustain ongoing engagement with the intervention. The TPB emphasizes the importance of perceived behavioral control; that is, engagement is more likely when parents feel the program allows them choice and control and is well matched to their/their child’s level of need. This is echoed by Sanders and Kirby (2012), who argue that a collaborative “consumer perspective,” which includes family input, is likely to be more responsive to families’ preferences and needs and will improve program reach.

Fourth, consideration might be given to using financial incentives for participation, although evidence on their effectiveness is mixed. A systematic review found that monetary incentives can increase enrolment in parenting programs and initial attendance rates but not retention (Gonzalez et al., 2018). Gross and Bettencourt (2019) found that 71% of low-income parents in their study cited cash incentives as a motivation to enroll, and that attendance rates of those parents were higher, but the quality of participation was unrelated to whether incentives motivated enrolment. Rodriguez et al. (2020) found that parents in their monetary incentive condition were more engaged in sessions than those in the program-as-usual condition but not more inclined to enroll. Ethical and practical concerns about financial incentives have also been raised—for instance, that they (i) undermine intrinsic motivation such that behavior change is not sustained, (ii) will be used irresponsibly (e.g., to buy cigarettes or alcohol), (iii) discriminate against parents who engage without needing incentives, (iv) use limited resources that could be better deployed, and (v) are unacceptable to practitioners in real-world service contexts (Gonzalez et al., 2018; Gross & Bettencourt, 2019). There is evidence to counter some of these; for example, parents in Gross and Bettencourt’s (2019) study reported that their primary motivation for participating was wanting to be a better parent and that they used the money to purchase groceries and items for their children. If incentives are considered, careful thought should be given to how these might be best used in terms of amount, frequency, format, and timing.

### Strengths and Limitations

To our knowledge, this is the first analysis of individual participant level data, pooled across multiple trials, of the social gradient of (parenting) program attendance; the focus on intervention engagement as the dependent variable is novel and extends the field’s understanding of the factors underlying differential benefit. We were able to reliably estimate the differences in attendance rates due to the large sample size of the pooled dataset and strong variation in markers of SES. A further strength was the broad conceptualization of SES, including indicators of income and employment, educational level, and sociodemographic status (i.e., lone and teen parent), which allowed us to identify differences in their relative importance for program engagement. While tests for contextual effects were underpowered, it was striking that there was no consistent pattern across models as there was for within-trial relationships. Finally, by pooling data across trials, our findings are likely to be generalizable across multiple service contexts and countries in Europe. That said, these data are limited by the extent to which it was possible to control for confounders of program attendance at the trial level or harmonize indicators across trials and countries. For example, low income was operationalized differently and may reflect differences in experiences of relative poverty. Moreover, although data on SES was fairly complete both across and within trials (Online Resource Table S2), attendance rates were missing in one trial (#2) and for some of the families within other trials. Using binary logistic regression, the original pooling study identified only missing data on unemployment as somewhat selective, with lone parent status predicting missingness (Gardner et al., 2017). There was no other evidence for selective missingness but we cannot be certain that missing data at the individual family level is not selective. Finally, we were only able to operationalize program engagement as relative dosage/attendance; future research should consider ways to reliably explore differences by SES in program reach and access as well as responsiveness to intervention content/processes.

### Conclusion

Our analyses suggest that there is a strong social gradient to parenting program attendance, even though program benefits seem to be comparable across SES. While it is possible that the measures used currently by programs to improve the engagement of all families have the potential to boost the attendance and consequent benefits for low-SES families—a rising tide may lift all boats—more research and intervention development is needed to understand and target specific factors that promote the engagement of socially disadvantaged families.

### Supplementary Information

Below is the link to the electronic supplementary material.

Supplementary file1 (DOCX 23 KB)

## Footnotes

1 Proportionate universalism refers to actions that are universal but with an intensity and a scale that is proportional to the level of disadvantage (Marmot Review Team, 2010, p. 15).

2 We include here only trials that had organized transport available to any/all families who needed it. Some studies were able to be responsive in emergencies to families who needed transport on particular occasions.
