# Supplementary material for: Does Social and Economic Disadvantage Predict Lower Engagement with Parenting Interventions? An Integrative Analysis Using Individual Participant Data
Source: Prev Sci. 2022 Jul 23;24(8):1447–58. doi: 10.1007/s11121-022-01404-1 (PMC10678811; doi:10.1007/s11121-022-01404-1)
Supplement: Supplementary file 1 — Supplementary file1 (DOCX 23 KB) [file 11121_2022_1404_MOESM1_ESM.docx]

**Table S1. Correlations between indicators of family socioeconomic status**

|  | Low income | Low education | Lone parenthood | Teen parenthood |
| --- | --- | --- | --- | --- |
| Low income |  |  |  |  |
| Low education | .287 |  |  |  |
| Lone parenthood | .339 | .140 |  |  |
| Teen parenthood | .178 | .150 | .202 |  |
| Unemployment | .555 | .285 | .479 | .243 |

*Note*. All correlations are significant at the 0.001 level.

**Table S2. Percentage of Complete Data Within and Across Trials**

|  | Low income | Low education | Lone parenthood | Teen parenthood | Unemployment | Attendance |
| --- | --- | --- | --- | --- | --- | --- |
| Trial #1 | 89% | 98% | 87% | 96% | 100% | 94% |
| Trial #2 | 92% | 92% | 92% | 87% | 100% | 0% |
| Trial #3 | 100% | 100% | 100% | 97% | 94% | 97% |
| Trial #4 | 99% | 95% | 100% | 100% | 99% | 99% |
| Trial #5 | 100% | 100% | 100% | 92% | 68% | 100% |
| Trial #6 | 100% | 78% | 100% | 100% | 0% | 61% |
| Trial #7 | 100% | 99% | 100% | 100% | 0% | 100% |
| Trial #8 | 100% | 100% | 100% | 97% | 86% | 97% |
| Trial #9 | 99% | 99% | 99% | 98% | 100% | 100% |
| Trial #10 | 95% | 98% | 98% | 98% | 100% | 100% |
| Trial #11 | 100% | 97% | 97% | 93% | 94% | 100% |
| Trial #12 | 90% | 87% | 98% | 94% | 95% | 100% |
| Trial #13 | 98% | 98% | 100% | 95% | 90% | 92% |
| Trial #14 | 76% | 87% | 69% | 90% | 97% | 84% |
| **Total** | **96%** | **94%** | **96%** | **96%** | **75%** | **91%** |
